# RNASEK interacting with PEDV structural proteins facilitates virus entry via clathrin-mediated endocytosis

**DOI:** 10.1128/jvi.01760-24

**Published:** 2025-01-21

**Authors:** Wenzhen Qin, Ning Kong, Shengsong Xie, Hailong Liu, Xinyu Yang, Yahe Wang, Xinyu Cao, Yuchang Liu, Jiarui Wang, He Sun, Wu Tong, Hai Yu, Hao Zheng, Wen Zhang, Guangzhi Tong, Tongling Shan

**Affiliations:** 1 Shanghai Veterinary Research Institute, Chinese Academy of Agricultural Sciences 118161 , Shanghai, China; 2 Jiangsu Co-Innovation Center for the Prevention and Control of Important Animal Infectious Disease and Zoonoses, Yangzhou University 38043 , Yangzhou, China; 3 Key Laboratory of Agricultural Animal Genetics, Breeding and Reproduction of the Ministry of Education, Key Lab of Swine Genetics and Breeding of Ministry of Agriculture and Rural Affairs, Huazhong Agricultural University 665711 , Wuhan, China; 4 School of Medicine, Jiangsu University 191611 , Zhenjiang, China; University of Kentucky College of Medicine, Lexington, Kentucky, USA

**Keywords:** PEDV, RNASEK, virus entry, endocytosis

## Abstract

Porcine epidemic diarrhea virus (PEDV), as a type of Alphacoronavirus causing acute diarrhea and high death rate among sucking piglets, poses great financial damage to the swine industry. Nevertheless, the molecular mechanism whereby PEDV enters host cells is unclear, limiting the development of PED vaccines and anti-PEDV agents. The present study found that the host protein ribonuclease kappa (RNASEK) was regulated by USF2, a transcription factor, and facilitated the PEDV replication. RNASEK was identified as a novel binding partner of PEDV, which interacted with a spike (S), envelope (E), and membrane (M) proteins on PEDV virion surfaces to increase the uptake not for attachment of PEDV virions. PEDV enters cells through the endocytosis pathways. RNASEK knockdown or RNASEK knockout assay revealed that through clathrin-mediated endocytosis (CME), RNASEK promoted the internalization of PEDV virions. Clathrin and the adaptor protein EPS15 only interacted with PEDV E protein, demonstrating that the RNASEK could target more virions through interaction with PEDV S, E, and M proteins to clathrin and EPS15 proteins rather than merely interacting with PEDV E protein to mediate the PEDV entry through CME. Moreover, our findings suggest that RNASEK, a newly identified host-entry factor, facilitates PEDV internalization by increasing the interaction of PEDV virions and EPS15-clathrin complex and may also provide a potential target for anti-PEDV therapies.

**IMPORTANCE:**

PEDV is the causative pathogen of porcine diarrhea, which is a highly infectious acute intestinal condition, that poses significant economic damage to the swine industry. However, the existing PED vaccines fail to provide adequate protection for piglets against PEDV infection. Although PEDV replication in cells has been widely described, the mechanisms beneath PEDV entry of the host cells are incompletely understood. In this study, we showed that RNASEK, regulated by the transcription factor USF2, is a new host factor increasing PEDV infection in LLC-PK1 cells. RNASEK can bind to multiple structural proteins of PEDV (S, E, and M proteins), therefore increasing the interaction between PEDV virions, clathrin, and EPS15 to promote PEDV virion entry. Apart from unraveling the entry mechanisms of PEDV, our findings also contributed to facilitating the development of anti-PEDV agents and PED vaccines.

## INTRODUCTION

Porcine epidemic diarrhea (PED), as a highly infectious swine intestinal condition that is induced by the PED virus (PEDV), typically manifests itself as vomiting and severe diarrhea in the clinical setting. In addition, PEDV can infect any age of pigs, while it is especially lethal in sucking piglets, with mortality up to 100% (1). The primary method of PED transmission is through the fecal–oral route and can spread through the fecal–nasal route as airborne particles (2, 3). Initially reported in the UK in the 1970s, PED spreads to the majority of pig-farming nations, which include various European nations and Asia countries (4, 5). As an endemic disease, it was not viewed as a worldwide concern. Until 2010, the highly pathogenic PEDV variants have appeared in China, quickly propagating across Asia, Europe, and American countries (6–10). However, existing PED vaccines have failed to provide sufficient protection for piglets against the PEDV variant infection.

PEDV, as a type of *Alphacoronavirus* (α-CoV) from the *Coronaviridae* family, possesses a single-stranded positive-sense ~28 kb RNA genome, encoding 16 nonstructural proteins (nsp1–nsp16), four structural proteins, namely spike (S), envelope (E), membrane (M), and nucleocapsid (N), as well as an accessory protein ORF3 (11). The S protein, which is vital for the binding and intracellular entry of PEDV by attaching to cell receptors and facilitating membrane fusion, is cleaved into the N-terminal S1 subunit and the C-terminal S2 subunit. The former encompasses the receptor-binding domain, while the latter is responsible for membrane fusion (12, 13). As the smallest structural protein of PEDV, the E protein plays a vital role in viral assembly and release, inhibiting the secretion of both type I and III interferons (IFNs) (14–16). The M protein is essential for viral envelope formation, which hinders type I IFN production (17). The N protein, a highly conserved phosphoprotein, is involved not only in viral replication, assembly, and budding but also in inhibiting type I IFN production and degrading host antiviral proteins to stimulate the proliferation of PEDV (18–20).

During virus infection, a very intricate process, the virions first attach to the receptors on cellular membranes and then internalize into the target cells through different pathways (21). Most coronavirus viruses, including transmissible gastroenteritis virus (TGEV) and severe acute respiratory syndrome coronavirus 2 (SARS CoV-2), enter cells through cellular endocytosis mediated by clathrin, caveolae, lipid rafts, or through macropinocytosis, etc. (22, 23). Among them, clathrin-mediated endocytosis (CME) is a widely recognized common endocytic pathway used by viruses, where a virus initially binds to cell surface receptors, and is subsequently packaged by clathrin-coated pits (CCPs) for migration into clathrin-coated vesicles (CCVs). In CCVs, the viral particles are transported to the early and late endosomes for viral genome release to achieve replication (21, 24, 25). Following the interaction between viral S protein and receptors, coronaviruses enter cells through a variety of endocytic pathways, followed by the endocytic process initiation. Although the COS7 and HepG2 cell entry of SARS-CoV uses the clathrin-mediated pathway, its Vero E6 cell entry uses the lipid raft pathway (26–28). Murine hepatitis virus strain A59 (MHV-A59) entry into murine fibroblast cells is mainly achieved by endocytosis through clathrin (29). PEDV enters Vero and IPEC-J2 cells via endocytosis mediated by caveolae, lipid rafts, and clathrin, and is transported through the endo-/lysosome pathway (21, 30). Despite the existing reports concerning the invasion mechanism of PEDV, its invasion strategy needs to be fully clarified.

The N- and C-termini of ribonuclease kappa (RNASEK), a dual-pass transmembrane protein, are apparently located intracellularly (31, 32). Although RNASEK exhibits a 100% amino acid homology among mice, rabbits, and humans, with ubiquitous expression and high conservation across mammals, its function in cellular remains unclear (31). RNASEK is essential for maintaining the levels of both plasma membrane-associated and cell-wide vacuolar ATPase (V-ATPase) compartments. The recent evidence has indicated the implication of RNASEK in the endosomal pathway. Its depletion leads to the weakening of CME and non-clathrin-dependent endocytosis, and thus the CCPs formed are enlarged. Although RNASEK is essential for various viruses, including dengue, influenza A, and rhinovirus, which depend on CME for entry, its role in general endocytic uptake is dispensable (32). RNASEK is not required for attachment, whereas it is essential for the uptake of such acid-dependent viruses as Rift Valley Fever, Sindbis, and West Nile (33). Moreover, whether RNASEK is implicated in the virion internalization process of PEDV and how it promotes the internalization of PEDV virions are still unclear.

It is indicated in this study that the host protein RNASEK was regulated by USF2, a transcription factor, aiming to promote PEDV replication. Subsequently, we found that RNASEK is a novel binding partner for PEDV, which was partially localized on cell surfaces and involved in the PEDV internalization. In addition, we demonstrated the interactions of RNASEK with the viral S2, E, and M proteins and clathrin protein. Afterward, only the E protein was involved in the interaction with the adaptor protein EPS15, which facilitated the E-EPS15-clathrin complex formation, causing PEDV endocytosis in LLC-PK1 cells. Our findings showed the specific mechanism beneath PEDV internalization and provided novel insights into the early phases of the CoV life cycle.

## RESULTS

### Transcription factor USF2 regulates RNASEK expression during PEDV infection

**Fig 1:**
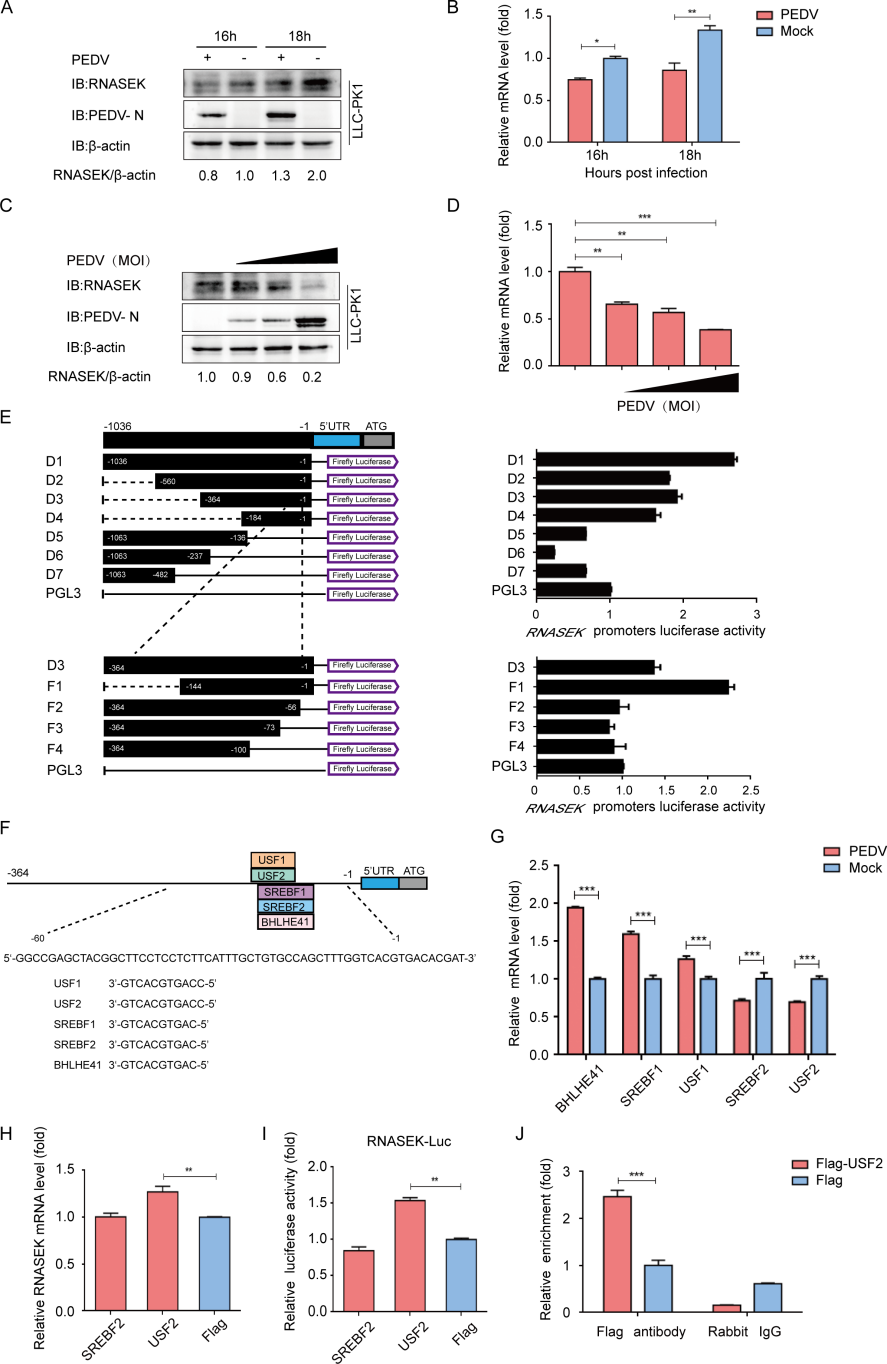
PEDV infection causes USF2-mediated downregulation of RNASEK expression. (**A and B**) After PEDV infection (MOI = 1), the LLC-PK1 cells were gathered at 16 and 18 hpi. RNASEK expression was assessed by Western blotting in combination with qRT-PCR. β-actin was used as the sample loading control. (**C and D**) At 16 hpi, the LLC-PK1 cells challenged with varying MOIs of PEDV were harvested. Protein and mRNA levels of RNASEK were investigated through western blotting and qRT-PCR assays. (**E**) For the dual luciferase assay, truncated RNASEK promoter constructs comprising Renilla luciferase reporter vector (pRL-TK-luc) were transfected into HEK 293T cells. (**F**) Based on the JASPAR vertebrate database, the TFBS of the RNASEK promoter was predicted. (**G**) To assess the relative mRNA levels for predicted genes, qRT-PCR was performed on PEDV-infected LLC-PK1 cells. (**H**) The plasmid encoding Flag-SREBF2 or Flag-USF2 was transfected into LLC-PK1 cells, and the lysate was subjected to qRT-PCR analysis. (**I**) For the dual-luciferase activity evaluation, HEK 293T cells were transfected with RNASEK promoter-driven luciferase vector and SREBF2- or USF2-encoding plasmids. (**J**) For ChIP analysis, Flag-USF2 plasmid was transfected into LLC-PK1 cells. Data presented are indicated to be means ± SDs from triplicate samples. *P < 0.05; **P < 0.01; ***P < 0.001.

To investigate the effects of PEDV infection on RNASEK expression, the protein and mRNA levels of RNASEK during virus infection were assessed. PEDV (strain JS-2013) is used to infect porcine renal proximal tubule epithelial cell-derived LLC-PK1 cells by setting the multiplicity of infection (MOI) to 1, according to the previous description (34). After the collection of the infected cells, western blotting and quantitative real-time PCR (qRT-PCR) were performed to evaluate the RNASEK expression at indicated hour post-virus infection (hpi). It was found that compared with the mock-infected cells, the PEDV-infected LLC-PK1 cells showed signiﬁcant downregulation of both the RNASEK protein and mRNA levels (Fig. 1A and B). The results were demonstrated with different MOI of PEDV infected with LLC-PK1 cells (Fig. 1C and D). Those ﬁnding suggested that PEDV infection downregulated the endogenous expression of RNASEK in host cells.

Then, to further clarify the transcriptional regulation of RNASEK, the 1,036 bp of RNASEK promoter along with its truncated sequences (D1–D7) were cloned into the pGL3-basic luciferase vector. Our finding indicated that luciferase expression was induced by the truncated sequences encompassing nucleotides from −136 to −1 (D1, D2, D3, and D4), suggesting that the core region of RNASEK promoter spans positions −136 to −1 (Fig. 1E). The promoter sequence was further truncated to determine the boundaries of minimal RNASEK core promoter. Based on the luciferase reporter assay result, the location of RNASEK core promoter lies within –56 to –1 (Fig. 1E). Besides, the potential transcription factor binding sites (TFBSs) for RNASEK promoter were determined using the JASPAR vertebrate database (http://jaspar.genereg.net/). It was found that the core region of the RNASEK promoter contained five potential transcription factor binding sites, including USF1-, USF2-, SREBF1-, SREBF2-, and BHLHE41-binding sites (Fig. 1F).

Subsequently, we subjected putative transcription factors to the qRT-PCR for mRNA quantitation, finding downregulation of SREBF2 and USF2 only during PEDV infection, which conformed to the expression of RNASEK in the PEDV infection process (Fig. 1G). To identify which transcription factor is implicated in the RNASEK expression regulation, cells were transfected with SREBF2- or USF2-expressing vectors. Based on the qRT-PCR and dual luciferase reporter results, only the USF2 protein expression could enhance RNASEK expression (Fig. 1H and I). Direct binding of USF2 to the core promoter region of RNASEK was demonstrated by the chromatin immunoprecipitation (ChIP) assay (Fig. 1J). These results suggested that during PEDV infection, the RNASEK expression was downregulated through USF2.

### RNASEK promotes PEDV infection in LLC-PK1 cells

**Fig 2:**
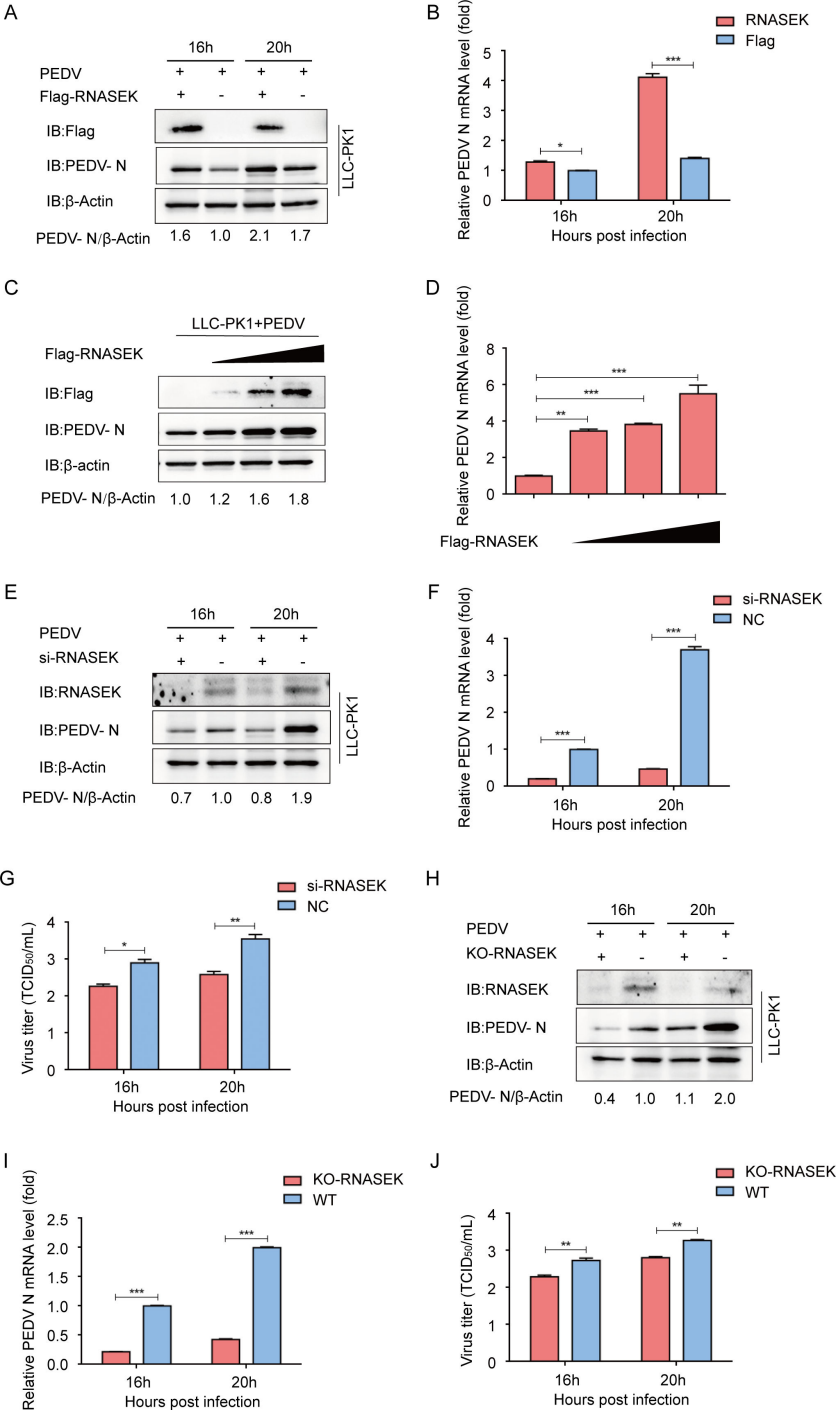
RNASEK promotes PEDV replication. (**A and B**) After transfection with the Flag-RNASEK encoding plasmid, the LLC-PK1 cells were challenged with PEDV (MOI = 1). Supernatants and cell lysates were performed by Western blotting and qRT-PCR. β-actin was used as the sample loading control. (**C and D**) After transfection with different concentrations of Flag-RNASEK plasmids, the LLC-PK1 cells were subjected to a PEDV challenge (MOI = 1). The samples were collected for western blotting and qRT-PCR. (**E–G**) After transfection with RNASEK siRNA, the LLC-PK1 cells were subjected to a PEDV challenge (MOI = 1). Sample analysis was performed through western blotting, qRT-PCR, and TCID_50_ assays. (**H-J**) PEDV replication in RNASEK KO LLC-PK1 cells was examined by Western blotting, qRT-PCR, and TCID_50_ assays. Data presented are suggested to be means ± SDs from triplicate samples. *P < 0.05; **P < 0.01; ***P < 0.001.

To investigate the role of RNASEK in PEDV infection, we transfected LLC-PK1 cells with RNASEK (Flag-RNASEK)-coding plasmids and then subjected them to PEDV challenge by setting MOI to 1. At 16 and 20 hpi, cells and supernatants were collected from the infected cultures. Western blotting and qRT-PCR indicated that RNASEK significantly facilitated PEDV replication in LLC-PK1 cells (Fig. 2A and B). It was also observed that RNASEK dose-responsively promoted PEDV replication (Fig. 2C and D). To verify the obtained results, RNASEK-targeting siRNAs were synthesized and selected. The viral yield was measured through western blotting, qRT-PCR, and TCID_50_ after transfecting LLC-PK1 cells with RNASEK siRNA and challenging them with PEDV by setting MOI to 1. PEDV replication in LLC-PK1 cells was inhibited after silencing RNASEK expression (Fig. 2E through G). To prove the role of RNASEK in PEDV infection, the RNASEK gene was knocked out in the LLC-PK1 cells using the CRISPR/Cas9 system, showing that the RNASEK deletion prominently reduced the replication of PEDV (Fig. 2H through J). These findings revealed that RNASEK exerts a crucial role in PEDV infection.

### RNASEK interacted with the S2, M, and E proteins of PEDV

**Fig 3:**
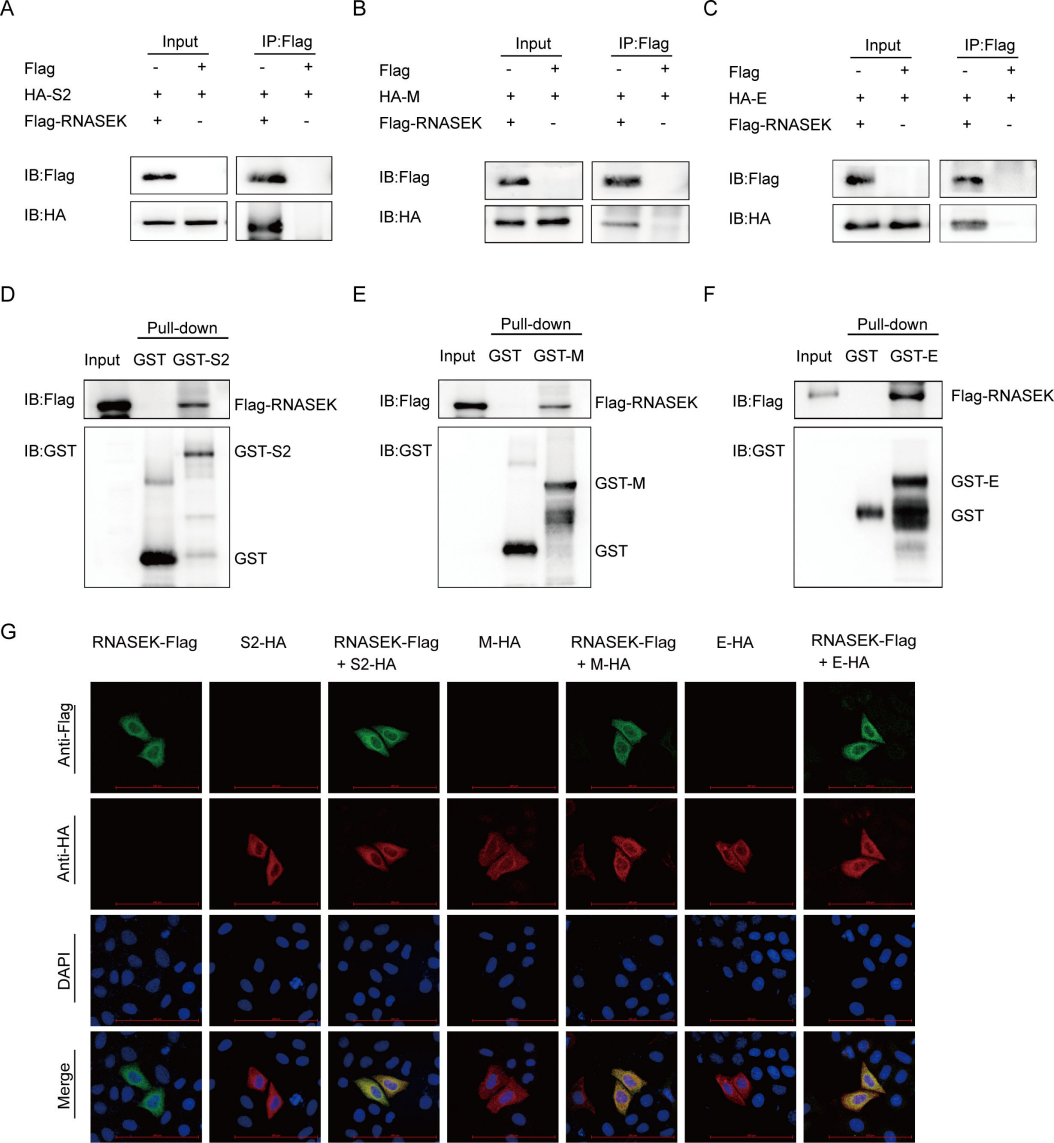
RNASEK interacted with the S2, M, E, and proteins of PEDV. (**A–C**) After co-transfection of HEK 293T cells with plasmids encoding Flag-RNASEK and HA-S2, HA-M, or HA-E, the associations between RNASEK and S2, M, and E proteins were assayed by Co-IP. (**D–F**) GST-S2, GST-M, GST-E, and RNASEK proteins were represented in bacterial strain BL21 (DE3). Meanwhile, the interactions of RNASEK with S2, M, and E proteins were investigated by GST pulldown assay. (**G**) After co-transfection of HeLa cells with plasmids encoding Flag-RNASEK and HA-S2, HA-M, or HA-E, the cells were exposed to incubation with designated primary antibody and fluorescently labeled secondary antibody, followed by results under confocal immunofluorescence microscopy. Scale bar, 100 µm.

To determine the molecular mechanisms of how RNASEK promotes PEDV replication, the associations between RNASEK and the viral S1, S2, E, M, and N proteins were assessed. After cotransfecting plasmids coding PEDV structural proteins and Flag-RNASEK into HEK 293T cells, the associations were examined using coimmunoprecipitation (Co-IP). Effective coimmunoprecipitation of RNASEK with S2, M, and E proteins was observed (Fig. 3A through C). In addition to the Co-IP assay, the glutathione *S*-transferase (GST) pulldown assay was used for validating the binding of PEDV S2, M, and E proteins to RNASEK. The results suggested that the GST fused to S2 protein (GST-S2), to M protein (GST-M), and to E protein (GST-E) bound to RNASEK, while the GST protein did not (Fig. 3D through F). This suggested direct binding of PEDV S2, M, and E proteins to RNASEK. Subsequently, confocal microscopy was used to examine the colocalization of RNASEK and S2, M, and E proteins. After cotransfecting HeLa cells with plasmids encoding Flag-RNASEK and HA-S2, HA-M, or HA-E, a confocal immunofluorescence assay was performed to assess protein localization. The result revealed cytoplasmic colocalization of RNASEK and S2, M, and E proteins (Fig. 3G). The above findings demonstrated that RNASEK interacted with the S2, M, and E proteins of PEDV.

### RNASEK is involved in PEDV internalization

**Fig 4:**
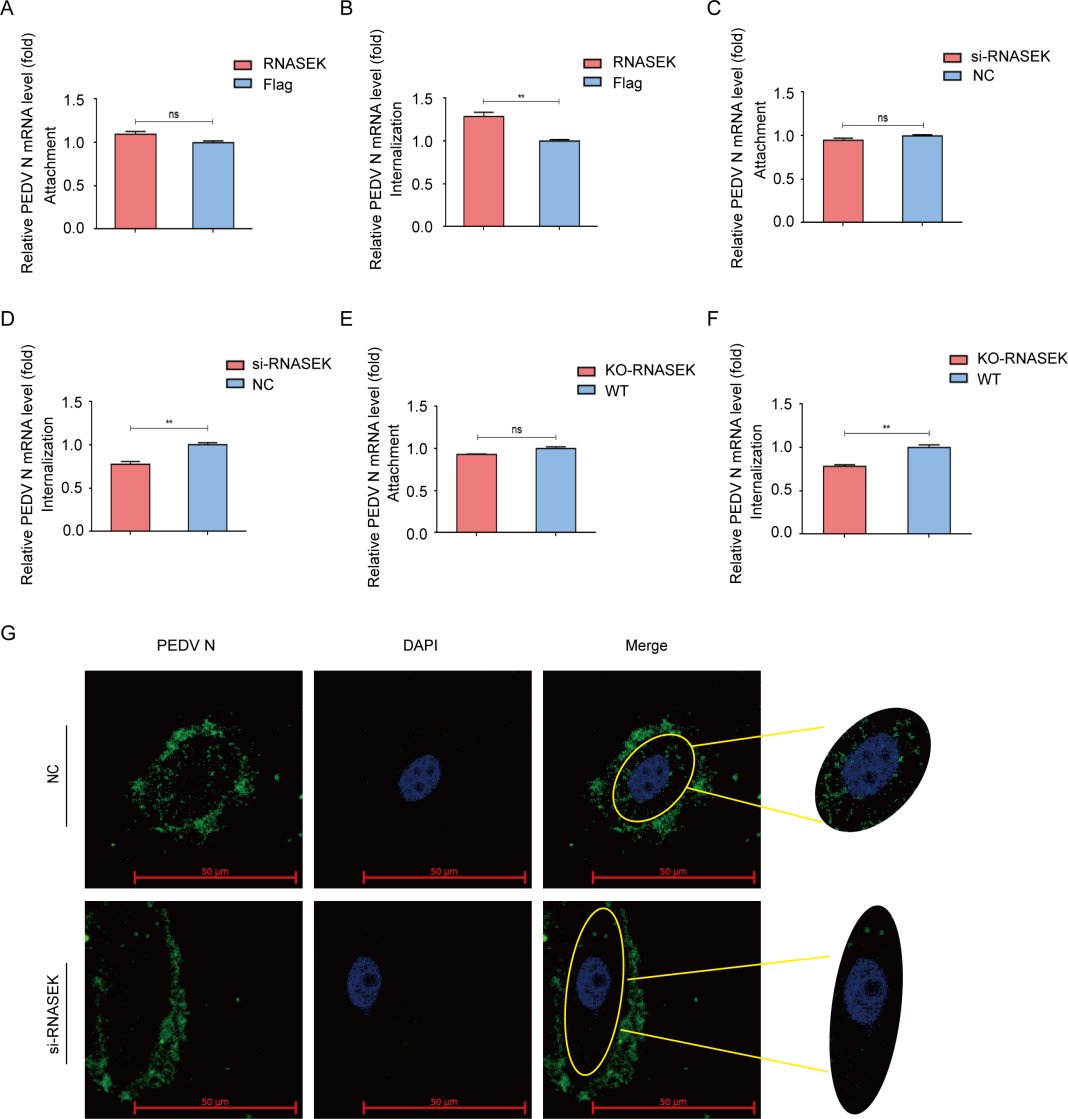
RNASEK is involved in PEDV internalization. (**A and C**) After transfection with a plasmid coding RNASEK or RNASEK siRNA, the LLC-PK1 cells were subjected to a PEDV challenge (MOI = 1). After 60 min of viral attachment at 4°C, the virus RNA copies of attachment were assessed through qRT-PCR. (**B and D**) Following transfection with plasmid-coding RNASEK or RNASEK siRNA, the LLC-PK1 cells were subjected to a PEDV challenge (MOI = 1). Following 60 min of viral attachment at 4°C, the cells were washed three times in prechilled PBS and subsequently transferred to 37°C. At 90 min after infection, the cells were washed in pH 3.0 citric acid buffer and explored by qRT-PCR. (**E and F**) Attachment and internalization of PEDV in RNASEK KO LLC-PK1 cells were performed by qRT-PCR. (**G**) LLC-PK1 cells were subjected to RNASEK siRNA transfection, followed by a PEDV challenge. Immunoﬂuorescence assay was employed to explore the internalization of PEDV. Scale bar, 50 µm.

The implication of RNASEK in the internalization of a diverse panel of viruses has been reported (33), and RNASEK interacted with most of the PEDV structural proteins (S2, M, and E proteins). Therefore, it could be speculated that RNASEK was probably implicated in the early phase of PEDV infection. To demonstrate this assumption, LLC-PK1 cells were transfected with RNASEK plasmid and then challenged with PEDV. As shown in Fig. 4A and B, although attached virions did not increase during PEDV infection, internalized virions increased significantly in RNASEK overexpression cells. Next, we synthesized the siRNAs targeting RNASEK and transfected them into LLC-PK1 cells. Based on the results, although attached virions did not decrease during PEDV entry (Fig. 4C), internalized virions decreased significantly in LLC-PK1 cells transfected with si-RNASEK (Fig. 4D). To further confirm the role of RNASEK, the RNASEK gene in LLC-PK1 cells was knocked out using CRISPR/Cas9 technology. qRT-PCR results showed that the RNASEK deletion significantly reduced virion internalization rather than attachment (Fig. 4E and F). In addition, we also surveilled the PEDV virion internalization at 90 min post-PEDV infection by high-magnification confocal microscopy. PEDV virions were internalized into the LLC-PK1 cells transfected with scrambled siRNA (NC), which formed speckled patterns. By contrast, si-RNASEK-transfected LLC-PK1 exhibited a lower PEDV internalization frequency (Fig. 4G). These results suggested an important role exerted by RNASEK in the PEDV virion internalization.

### RNASEK-mediated PEDV internalization is clathrin dependent

**Fig 5:**
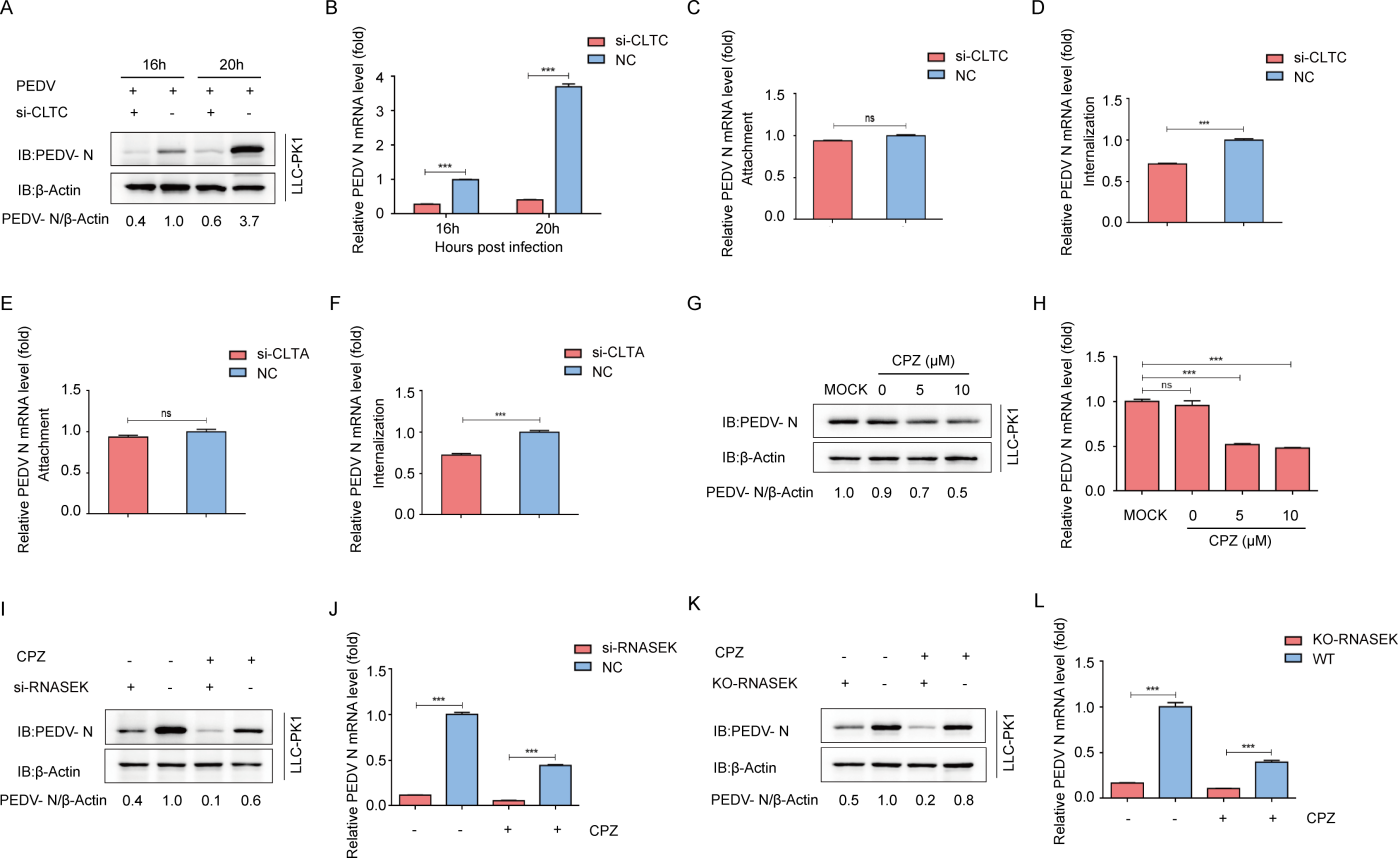
RNASEK-mediated PEDV internalization is clathrin dependent. (**A and B**) Following 24 h of CLTC siRNA transfection, the LLC-PK1 cells were subjected to PEDV challenge and assayed by western blotting and qRT-PCR. (**C-F**) After 24 h of si-CLTC or si-CLTA transfection, the LLC-PK1 cells were subjected to a PEDV challenge (MOI = 1). Viral attachment and internalization assays were performed by qRT-PCR. (**G and H**) After 60 min of incubation with varying concentrations of CPZ, the LLC-PK1 cells were challenged with PEDV. Western blotting and qRT-PCR were conducted to assess the genomic DNA copies of PEDV in these cells. (**I and J**) After 1 h of transfection with si-RNASEK or pretreatment with 5 µM of CPZ at 37°C, the LLC-PK1 cells were incubated with PEDV, and subsequently assayed by qRT-PCR and western blotting. (**K and L**) After 60 min of pretreatment with 5 µM CPZ, the RNASEK KO LLC-PK1 cells were subjected to a PEDV challenge (MOI = 1). The viral genome level was examined by western blotting and qRT-PCR.

For a variety of viruses, CME is the most frequently involved internalization mechanism. A prior study has revealed the association of CME with Vero cell internalization of PEDV (21). Clathrin light and heavy chains (CLTA and CLTC), which form a triskelion shape, are a vital component in regulating the clathrin lattice formation and disassembly (35). To conﬁrm whether the infection of PEDV in the naturally susceptible cells (LLC-PK1 cells) was dependent on CME, siRNAs were transfected into LLC-PK1 cells to lower the CLTC expression, followed by the challenge of cells with PEDV. The viral protein and mRNA levels in PEDV-challenged cells all decreased significantly after CLTC knockdown (Fig. 5A and B), implying a vital mediator role of clathrin in the PEDV infection. To further verify clathrin’s function in the virus’s life cycle, attachment and internalization assays were conducted, finding that CLTC or CLTA knockdown exerted no significant influence on PEDV attachment (Fig. 5C and E), but decreased the PEDV internalization efﬁciency (Fig. 5D and F), indicating that clathrin involved in viral internalization rather than attachment during PEDV infection.

Chlorpromazine (CPZ) is a CME inhibitor capable of preventing the coated pit formation at the plasma membrane, as well as the clathrin lattice assembly on endosomal membranes (36). To further assess CME’s function in the PEDV infection process, this study used 0, 5, and 10 µM CPZ to pretreat the LLC-PK1 cells, finding that CPZ pretreatment significantly and dose-responsively blocked the PEDV infection (Fig. 5G and H). To examine whether CME is implicated in RNASEK-mediated PEDV entry, LLC-PK1 cells were treated with CPZ inhibitor after transfecting si-RNASEK into them, finding that the RNASEK knockdown and CPZ treatment displayed a resembling effect on PEDV internalization. The internalization of PEDV can be mediated by both RNASEK and CME. Both the RNASEK knockdown and the CPZ treatment exhibited synergistic inhibition against PEDV infection, implying that RNASEK could probably mediate PEDV internalization through CME (Fig. 5I and J). RNASEK knockout cells further corroborate the concept of CME involvement in RNASEK-mediated viral internalization (Fig. 5K and L). Therefore, it could be hypothesized that RNASEK might be involved in PEDV virion internalization via CME.

### The interaction between RNASEK, clathrin, and PEDV structural protein

**Fig 6:**
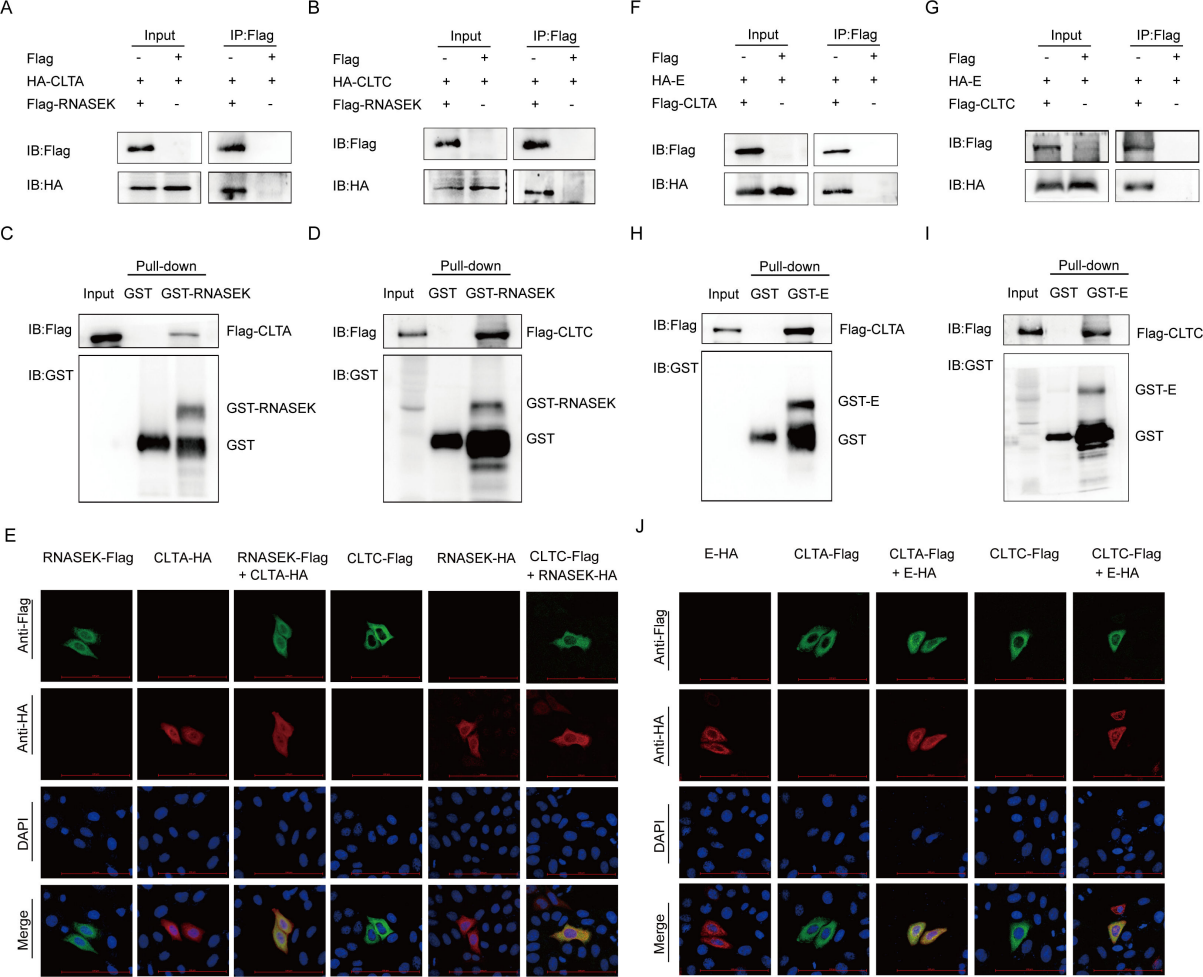
The interactions between RNASEK, clathrin, and PEDV structural proteins. (**A and B**) After transfection of HEK 293T cells with Flag-RNASEK and HA-CLTA or HA-CLTC encoding plasmids, a Co-IP assay was conducted by adopting anti-Flag binding beads, and the precipitated proteins were Western blotted. (**C and D**) The association of RNASEK with CLTA or CLTC was assessed by GST affinity–isolation assay. (**E**) After transfection of plasmids encoding RNASEK and CLTC or CLTA, the HeLa cells were labeled with designated primary and secondary antibodies. Nuclear staining was performed using DAPI, and fluorescence signals were identified by confocal immunofluorescent microscopy. Scale bars: 100 µm. (**F and G**) Plasmids encoding HA-E, Flag-CLTC, or Flag-CLTA were transfected into the HEK 293T cells. Co-IP assay was performed with anti-Flag-binding beads. (**H and I**) The interaction of PEDV E with CLTA or CLTC was examined through GST affinity–isolation assay. (**J**) After transfection with HA-E and Flag-CLTC or Flag-CLTA plasmids, the Hela cells were labeled with antibodies and assessed by confocal immunofluorescence microscopy. Scale bars: 100 µm.

To further identify the RNASEK-mediated PEDV virions internalization via CME, the association of RNASEK and clathrin (CLTC and CLTA) was examined. The Co-IP assay indicated coimmunoprecipitation of Flag-RNASEK with HA-CLTC and HA-CLTA (Fig. 6A and B). In addition, the correlation between RNASEK and clathrin was also demonstrated by the GST affinity–isolation assay, showing that RNASEK was directly bound to CLTC and CLTA (Fig. 6C and D). Confocal imaging also demonstrated the colocalization of RNASEK with CLTC and CLTA (Fig. 6E). Co-IP and GST affinity–isolation assays were performed to assess the interactions between PEDV structural protein (S1, S2, M, and E) and CLTC or CLTA. Only the coimmunoprecipitation of PEDV E protein with CLTA and CLTC was identified in HEK 293T cells (Fig. 6F and G). The GST affinity–isolation and confocal immunofluorescence assays also proved the interaction between PEDV E protein and CLTC or CLTA (Fig. 6H through J). The above results suggested that RNASEK, clathrin and PEDV E protein interacted with each other and that RNASEK regulated PEDV virions internalization through CME.

### The interaction of RNASEK and EPS15 is involved in PEDV internalization

**Fig 7:**
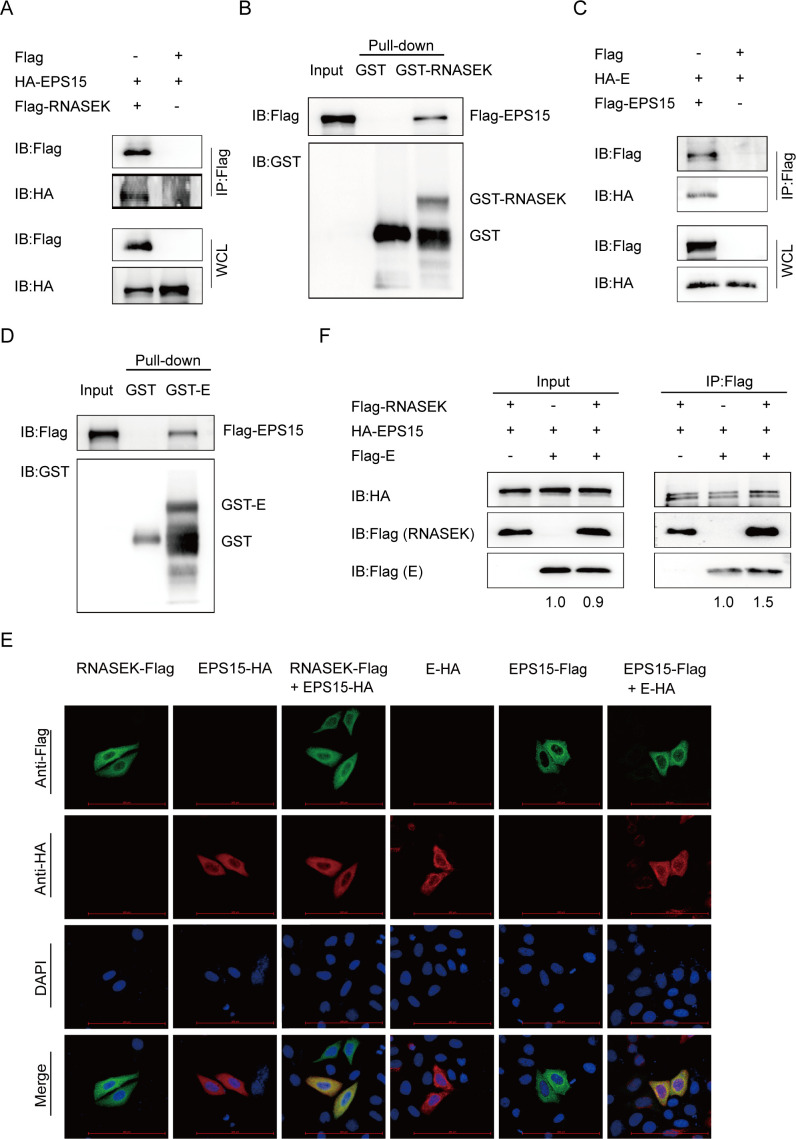
RNASEK interacts with EPS15 and is implicated in PEDV internalization via CME. (**A**) HEK 293T cells transfected with HA-EPS15 and Flag-RNASEK encoding plasmids were exposed to Co-IP assay. (**B**) The association of RNASEK with EPS15 was assessed through GST affinity–isolation assay. (**C**) After transfecting HA-E and Flag-EPS15 vectors into HEK 293T cells, the interaction of EPS15 with E was analyzed through Co-IP. (**D**) After indicating EPS15 and GST-E proteins in strain BL21 (DE3), they were subjected to GST pulldown assay. (**E**) After transfecting plasmids encoding EPS15 and RNASEK or E into HeLa cells, confocal immunofluorescence microscopy was used for the fluorescence signal observation. Scale bars: 100 µm. (**F**) Lysates of HEK 293T cells overexpressing RNASEK, EPS15, and E were immunoprecipitated with anti-Flag antibodies.

EPS15, a key component of CCPs interacting with the major clathrin adaptor protein complex 2 (AP-2), is implicated in the Vero cell internalization of PEDV virions through CME (21). To assess whether RNASEK associates with EPS15 to facilitate PEDV virion internalization, a Co-IP assay was initially conducted, discovering the association of EPS15 with RNASEK (Fig. 7A). We further investigated the interaction between EPS15 and RNASEK by GST affinity–isolation assay and immunofluorescence assay, finding that RNASEK directly bound with EPS15 (Fig. 7B and E). To further verify the correlations between EPS15 and PEDV S1, S2, M, N, and E proteins, Co-IP, GST pulldown and immunofluorescence assays were performed. The results demonstrated colocalization and immunoprecipitation of EPS15 only with PEDV E protein (Fig. 7C through E), suggesting that the RNASEK, EPS15, and PEDV E protein might be forming a complex for mediating the PEDV virion internalization. To verify the role of RNASEK protein in this complex, the plasmids coding RNASEK, EPS15, and PEDV E were transfected in HEK 293T cells, finding formation of a complex by the three proteins, where RNASEK promoted the interaction of EPS15 and PEDV E protein (Fig. 7F). These results suggested that RNASEK regulated the internalization of PEDV virions by acting as a mediator between EPS15 and PEDV E protein.

## DISCUSSION

**Fig 8:**
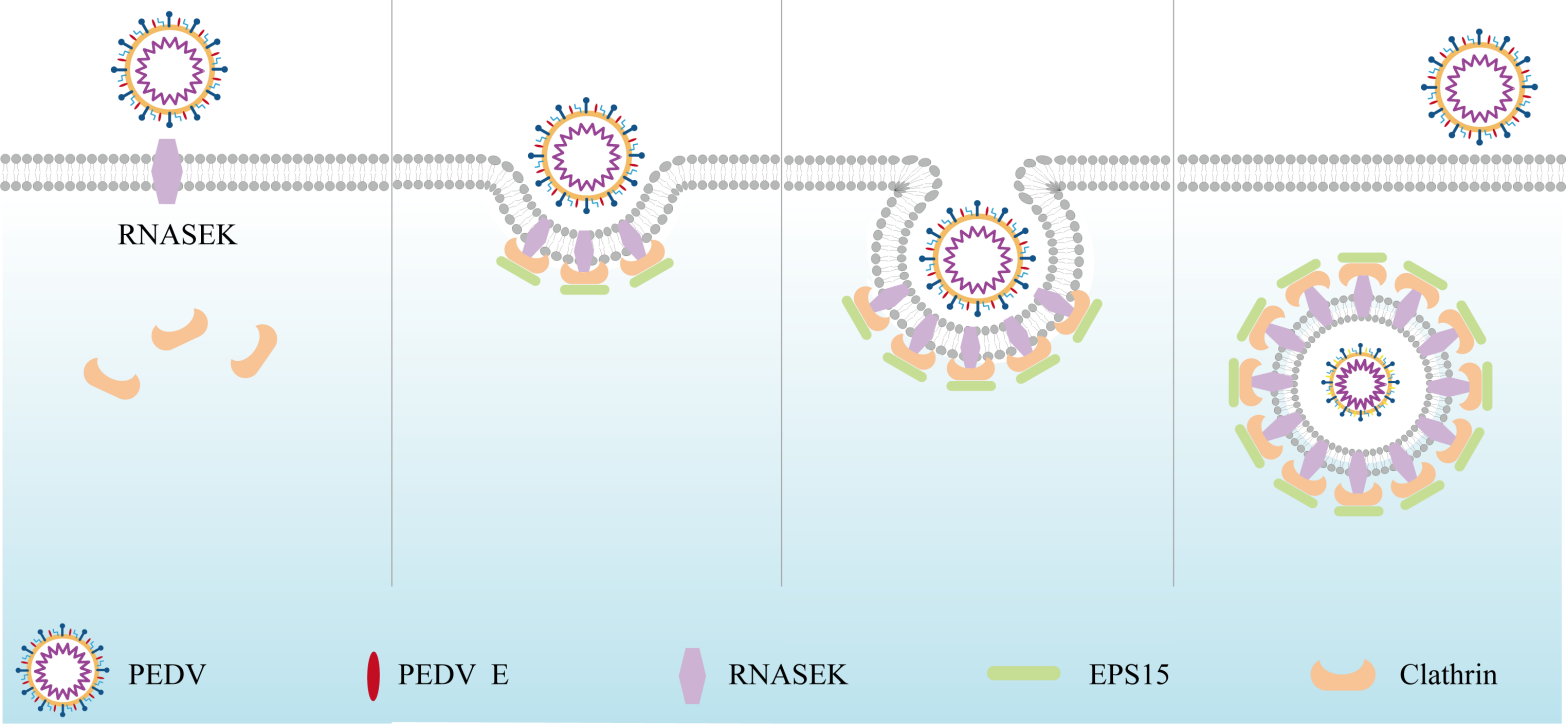
Schematic depiction of RNASEK in boosting PEDV endocytosis as a host factor. RNASEK interacted with PEDV S2, M, and E proteins. Subsequently, the signal was transmitted to clathrin and EPS15 to form the virions-RNASEK-EPS15 axis. This process promoted the EPS15-clathrin complex formation and aggregation, causing the endocytosis of PEDV virions in the infection process.

PED refers to a highly infectious acute intestinal condition induced by PEDV. With the occurrence of highly pathogenic variants, PEDV has posed huge economic damage to the worldwide swine industry. Considering our limited knowledge about the virus, particularly on the entry and replication mechanisms at the molecular level, no drug or vaccine is currently available for the control of PED effectively. PEDV, which parasitizes and proliferates intracellularly, achieves invasion, replication, and infectious particle release by exploiting diverse host factors (37, 38). In this study, the RNASEK expression in PEDV-infected cells was shown to be regulated by USF2. The interactions of S2, M, and E proteins of PEDV were identified with the RNASEK protein. Then, RNASEK interacted with EPS15 to promote the host cell internalization of PEDV virions through CME (Fig. 8).

RNASEK is also known as RNase K or RNase κ. The highly conserved trait, endonuclease activity, and universal expression of the RNASEK family members suggest their crucial roles during biological processes (39). Implication of RNASEK family in a variety of immune-associated diseases has been reported, including viral infections and malignancies (40, 41). In this study, RNASEK expression was correlated with the encouragement of PEDV infection. During the identification of possible transcription factors regulating the RNASEK expression, our results demonstrated that the minimal RNASEK core promoter region occurs at positions –56 to –1. The investigation of the RNASEK regulatory factors found that its expression is regulated by USF2.

Previous studies have demonstrated that internalization of a diverse panel of medically significant viruses (e.g., human rhinovirus, dengue, and influenza A) necessitates RNASEK (32, 33, 42). Loss of RNASEK leads to enlarged CCPs at the cellular surface and somewhat increased endo-lysosomal acidity, blocking the entry and replication of diverse viruses entering cells from endosomal compartments rather than at the plasma membranes (33). Consistently, it was demonstrated that RNASEK regulated PEDV replication. Through performing a few experiments, we attempted to show the molecular mechanisms beneath RNASEK-mediated viral replication. Importantly, the knockdown of RNASEK expression blocked PEDV infection remarkably, emphasizing its critical role in viral entry.

The essential initial step in viral infection is cell entry. While some viruses can enter the cytosol through direct penetration of cell membranes, most viruses utilize the endocytic pathways allowing cells to uptake growth factors, nutrients, hormones, and other extracellular substances (43). The host endocytic mechanisms most commonly utilized by viruses include caveolae/lipid raft-mediated endocytosis, CME, micropinocytosis, as well as CCIPs (36). Studies have indicated that CME provides an effective entry route for some coronaviruses, including Human Coronavirus NL63 (HCov-NL63) (44), TGEV (45), PEDV (21), SARS-CoV (26), SARS-CoV-2(22), MHV (46), porcine hemagglutinating encephalomyelitis virus (PHEV) (47), infectious bronchitis virus (IBV) (48), and porcine deltacoronavirus (PDCoV) (49). In this study, PEDV was shown to enter LLC-PK1 cells through the CME pathway, and deletion of RNASEK significantly affected its internalization, suggesting a crucial role played by RNASEK in the PEDV internalization process. Through treatment with CPZ inhibitor and siRNA interference, we also found that the PEDV entry necessitated CME and clathrin expression. Our findings suggest that RNASEK influences the LLC-PK1 cell internalization of PEDV through the CME pathway.

CME of viruses involves such steps as the attachment and binding through cell membrane receptors, signaling cascade initiation, virus-localized clathrin assembly, maturation, and CCV fission (36, 50). The association of clathrin with viral cargoes on the cellular membranes. Nevertheless, it relies on a variety of accessory proteins. Among these proteins, a group of adaptor proteins like AP-2, epsin, AP180, and Eps15 serve as a scaffold between the viral cargoes and the clathrin lattice (51, 52). In this study, it was indicated that RNASEK could target more virions through making interactions with PEDV S, E, and M proteins to clathrin and EPS15 proteins rather than merely interacting with PEDV E protein to mediate PEDV entry through CME. RNASEK, clathrin, and EPS15 synergistically facilitated the PEDV virion internalization through CME.

### Conclusions

In this study, RNASEK was identified as a novel host factor implicated in the PEDV internalization, finding that clathrin and EPS15 interacted with RNASEK in directing the PEDV invasion through the CME pathway. During this process, RNASEK interacted with PEDV S, E, and M proteins and carried more PEDV virions to clathrin and EPS15 protein complex which promoted PEDV internalization. On the whole, the present study highlights a novel mechanism for PEDV internalization into LLC-PK1 cells. Our findings broaden extant understanding of PEDV infection at early phases and provide a novel perspective for the development of anti-PEDV therapies.

## MATERIALS AND METHODS

### Antibodies and reagents

Anti-Flag M2 antibody (F1804) and chlorpromazine hydrochloride (CPZ) used were from Sigma-Aldrich products. Anti-GST antibody (10000-0-AP), anti-β-actin (66009-1-Ig), and anti-HA antibody (HRP-81290) were acquired from Proteintech Group. HRP-labeled anti-mouse IgG antibody (SB-AB0102) along with anti-rabbit IgG antibody (SB-AB0101) used were ShareBio products. Cross-adsorbed secondary antibodies, the goat anti-rabbit IgG (H + L) Alexa Fluor 488 (A-11008), and goat anti-mouse IgG (H + L) Alexa Fluor 594 (A-11032), were Invitrogen products. Anti-RNASEK was procured from Abmart. SiRNAs were constructed and synthesized by GenePharma (Table 1).

**TABLE 1:** Primer and siRNA sequences used in this study

Purpose	Name	Sequence (5′−3′)
Real-time PCR primers	PEDV *N* forward	GAGGGTGTTTTCTGGGTTG
	PEDV *N* reverse	CGTGAAGTAGGAGGTGTGTTAG
	*pRNASEK* forward	CCTGCGGCATCGTCCTTA
	*pRNASEK* reverse	TGAGCCGAACTTGGCAGAA
	*pUSF1* forward	CAGATTCAGGAAGGTGCGGT
	*pUSF1* reverse	CTGGGTCATGGATTGAGTGG
	*pSREBF1* forward	CTTTGCAGACCCTGGTGAGC
	*pSREBF1* reverse	GGCGGATTTATTCAGCTTCG
	*pUSF2* forward	CGTCCAGTGTGGGAGATACC
	*pUSF2* reverse	GCCTCCGCTCCACTTCATT
	*pSREBF2* forward	GTGCGTGCTCACTTTACCG
	*pSREBF2* reverse	AGGTAGCCCCGCTGACATT
	*pGAPDH* forward	ATGGATGACGATATTGCTGCGCTC
	*pGAPDH* reverse	TTCTCACGGTTGGCTTTGG
siRNA sequences	*si-RNASEK* sense	GCGUCGCUUCUGUGCUGUGTT
	*si-RNASEK* antisense	CACAGCACAGAAGCGACGCTT
	*si-CLTA* sense	GAGCCUGAAAGUAUCCGUATT
	*si-CLTA* antisense	UACGGAUACUUUCAGGCUCTT
	*si-CLTC* sense	CAGCUUGUAAGACUGGGCAGAUCAA
	*si-CLTC* antisense	UUGAUCUGCCCAGUCUUACAAGCUG
	NC sense	UUCUCCGAACGUGUCACGUTT
	NC antisense	ACGUGACACGUUCGGAGAATT

### Cell culture and virus

Human embryonic kidney 293T cells (CRL-11268) and African green monkey kidney (Vero) cells (CCL-81; both ATCC) were cultured in Dulbecco’s modified Eagle’s medium (DMEM) (12430-054; Gibco) containing 10% fetal bovine serum (FBS; 10099141; Gibco). Porcine kidney (LLC-PK1) cells, which were donated by Rui Luo (Huazhong Agricultural University), were cultured in modified Eagle’s medium (11095-080; Gibco). All the involved cells were cultivated at 37°C under the atmosphere covering 5% concentration of CO_2_. *In vitro* was kept in accordance with the previous description, and JS-2013 (a variant strain of PEDV) was isolated. PEDV was propagated and titrated using Vero cells following the former description (53).

### Plasmids and transfection

RNASEK- and other protein-encoding recombinant plasmids were subjected to homologous recombination cloning through the ClonExpress II one-step kit (C112-02; Vazyme Biotech). Upon reaching confluency of 80%–90%, the recombinant plasmids were transfected into HEK 293T or LLC-PK1 cells by adopting the Lipofectamine 3000 reagent (L3000-015; Invitrogen). The cells reaching confluency of approximately 50%–60% were transfected with siRNAs following the protocol of Lipofectamine RNAiMAX (13778-150; Invitrogen).

### RNA extraction and qRT-PCR

After washing in phosphate-buffered saline (PBS), the cells were lysed, followed by total RNA extraction via the RNeasy mini kit (RC112-01; Vazyme Biotech) following the protocol of the manufacturer. Subsequently, the extracted RNA was reversely transcribed into cDNA via the PrimeScript RT reagent kit (R323-01; Vazyme Biotech). qRT-PCR was performed following the protocol of SYBR Premix Ex Taq (q711-02; Vazyme Biotech).

### Virus binding and internalization assay

After 30 min of pre-chilling at 4°C, the LLC-PK1 cells were challenged with PEDV (MOI = 1), followed by being incubated for 60 min at 4°C. The unbound virus was eliminated by three PBS washes. For internalization assay, the dishes were overlaid with a complete medium and transferred to 37°C for 90 min to allow virus internalization followed attachment. To inactivate citric acid buffer viruses, the cells were subject to treatment for 2 min with pH 3 citric acid buffer at ambient temperature and then washed in PBS twice. The viral copy number was assessed by qRT-PCR.

### Western blotting

After washing in cold PBS, the cells were lysed by RIPA buffer (89901; Thermo Fisher). The proteins were isolated via SDS-PAGE and subsequently shifted onto nitrocellulose membranes (10600001; Cytiva) for the western blotting analysis. Thereafter, 60 min of membrane blockade was performed with dry skimmed milk (5%; 232100; BD) in 0.2% Tween 20-involving PBS (PBST). Afterward, the membranes were subjected to 60 min incubation using primary antibodies at room temperature and subsequently washed in PBST. In addition, the membranes were subjected to an extra 1 h incubation using HRP-conjugated secondary antibodies, followed by protein assays with the ECL (enhanced chemiluminescence) substrate (SB-WB004; ShareBio).

### Co-immunoprecipitation assay

Following 24 h of transfection with designated plasmids, the cells were lysed at 4°C using the NP40 buffer (FNN0021; Invitrogen). Subsequently, the lysates were subjected to centrifugation-based clearing with protein G (Dynabeads; 10004D; Invitrogen)-bound affine antibodies, followed by washing in 0.02% PBST. The following step was Dynabeads suspension in a pH 2.8 elution buffer (50 mM glycine). Besides, the proteins were assayed using indicated antibodies by immunoblotting.

### Dual-luciferase reporter assay

The 293T cells were transfected with a luciferase reporter plasmid (pRL-TK) and designated plasmid or the blank control plasmid through Lipofectamine 3000. After 24 h, cell lysis was performed and the Renilla and firefly luciferase activities were analyzed with a dual-luciferase reporter system (DL101-01; Vazyme Biotech) in accordance with the guidelines of the manufacturer.

### GST pulldown

Full-length indicated genes were cloned into the pCold GST plasmid (3372) or pCold TF plasmid (3365; Clontech Laboratories). BL21 competent cells (C504-03; Vazyme Biotech) were employed to express recombinant proteins. Following the protocol of the GST Protein Interaction Pull-Down kit (21516; Thermo Fisher), protein interplays were assessed. After eluting with reduced glutathione, the proteins were Western blotted.

### Fluorescence microscopy

After 24 h of cotransfection with indicated plasmids, the cells were fixed for 15 min in 4% paraformaldehyde (P6148; Sigma-Aldrich) and subsequently permeabilized for 10 min with 0.1% Triton X-100 (T9284; Sigma-Aldrich). After PBS washed three times, the cells were subject to incubation using a primary antibody, and further using a fluorescent dye-labeled secondary antibody, followed by the results with a confocal immunofluorescent microscope (Carl Zeiss).

### Statistical analysis

Data were processed by two-sided student’s *t*-test using Prism 6 (GraphPad Software) and indicated as means ± standard deviations (SDs). Differences were also considered to be of significance when *P* < 0.05.
